# Supplementary material for: Mechanical loading induces the longitudinal growth of muscle fibers via a rapamycin-insensitive mechanism
Source: Sci Adv. 2026 Feb 13;12(7):eaec5134. doi: 10.1126/sciadv.aec5134 (PMC12904167; doi:10.1126/sciadv.aec5134)
Supplement: Supplementary file 1 — Supplementary Text Figs. S1 to S10 Table S1 Legends for movies S1 and S2 Legend for data S1 [file sciadv.aec5134_sm.pdf]

Science Advances
AAAS

Supplementary Materials for

Mechanical loading induces the longitudinal growth of muscle fibers via a rapamycin-insensitive mechanism

Jamie E. Hibbert *et al.*

Corresponding author: Troy A. Hornberger, troy.hornberger@wisc.edu

Sci. Adv. **12**, eaec5134 (2026)
DOI: 10.1126/sciadv.aec5134

The PDF file includes:

Supplementary Text
Figs. S1 to S10
Table S1
Legends for movies S1 and S2
Legend for data S1

Other Supplementary Material for this manuscript includes the following:

Movies S1 and S2
Data S1

Supplementary Text:

Newly Synthesized Protein (NSP) Hot Spot Quantification Procedures

Due to the subjective nature of these analyses, the investigators acquiring the images, as well as those who performed the analyses, were blinded to the experimental condition of the samples.

NSP Hot Spot Positive Fibers

Fibers were categorized as positive for NSP hot spots if they contained multiple loci that were densely populated with NSPs. To illustrate this, four 63x images of NSPs in muscles that were subjected to mechanical overload or the sham condition are shown in Supplemental Figure S6.

Normal vs. Disarrayed ROIs

Fibers that were categorized as positive for NSP hot spots were screened for the presence of "normal" and "disarrayed" 100 μm^2 (100 x 100 pixels) regions of interest (ROIs) when viewing the signal for α-actinin. ROIs that were classified as "disarrayed" contained highly discontinuous Z-lines. When selecting these ROIs, preference was given to regions with the highest discontinuity and/or the appearance of numerous "Y" shaped splits (Fig. S7).

ROIs that were classified as "normal" contained continuous Z-lines. When selecting these ROIs, efforts were made to maximize the distance of the ROI from surrounding areas of disarray. For example, in Supplemental Figure S8, three "normal" ROIs (white boxes labeled A-C), as well as three "disarrayed" ROIs (lavender boxes labeled D-F) were selected, and the top-ranking "normal" ROI in this example is A, followed by C, and then B.

Fibers that contained both "normal" and "disarrayed" ROIs were subjected to further quantitative analyses. Specifically, an equivalent number of "normal" and "disarrayed" ROIs within each fiber were identified (up to 3 of each type of ROI per fiber). Then, within each ROI, the periphery of the individual hot spots was manually traced, and the total number of hot spots per ROI was recorded. Some fibers were permissive to the analysis of multiple ROIs and, in these instances, the mean values for the "normal" and "disarrayed" ROIs within the fiber were calculated (Fig. S9). In all cases, the final "normal" and "disarrayed" ROI data for each fiber were recorded as paired values. Notably, several of the fibers that were categorized as positive for NSP hot spots contained an extensive number of "disarrayed" ROIs but did not contain a "normal" ROI for a paired analysis, and for this reason, ROI level quantification on such fibers was not performed.

Classification of NSP Hot Spot Morphology

To be included in the classification analysis, an NSP hot spot needed to have a signal intensity at least 2-fold higher than the local background and a diameter between 400 and 1900 nm (corresponding to the values that represents the 0.5^{th} percentile of the minimal and 99.5^{th} percentile of the maximal Feret diameters of sarcomeres in plantaris muscles that had been subjected to 8 days of MOV; see Supplemental Figure S1). All fibers within a given 63x field that contained at least 15 qualifying NSP hot spots were analyzed. Within each of these fibers, at least 15 and up to 30 qualifying NSP hot spots were randomly selected and classified according to whether their morphology conformed to one of three previously proposed models of in-series sarcomerogenesis (*35, 38, 42*).

The first model we considered was based on a sarcomere transverse Z-line splitting event that was recently described by Rodier et al. (*42*). Specifically, this model proposes that in-series sarcomerogenesis is initiated when titin / thick filament complexes detach from the Z-lines of an existing sarcomere. Equal portions of these complexes are then pulled in opposite directions, and lead to the exposure of binding sites that facilitate the recruitment of new sarcomeric proteins. As illustrated in Figure 8A, if the recruited proteins consist of NSPs, then this process would give rise to exactly two in-series sarcomeres that are densely populated with NSPs. An example of this is shown in Fig. S10A, and all NSP hot spots that displayed this morphology were classified as Rodier et al. (*42*) Z-line splits.

The second model of in-series sarcomerogenesis that we considered was based on a sarcomere transverse Z-line splitting event originally described by Yu et al. (*35*). Here, the process is proposed to begin with the breakdown and broadening of a single Z-line. As this process continues, the remaining components of the Z-line get pulled in opposite directions, which allows new sarcomeric proteins to be incorporated into the region of expansion. As illustrated in Figure 8B, if these sarcomeric proteins are NSPs, then this process would result in the formation of a single sarcomere that is densely populated with NSPs. An example of an NSP hot spot whose morphology is consistent with this model is shown in Supplemental Figure S10B, and all such NSP hot spots were classified as Yu et al. (*35*) Z-line splits.

The final model of in-series sarcomerogenesis we considered was proposed by Jahromi and Charlton (*38*) and involves sarcomere transverse splitting at the H-zone. More precisely, the model argues that thick filaments are bisected at the H-zone, after which the two halves get pulled in opposite directions. As this occurs, it is thought that new myosin molecules get incorporated at the severed ends of the thick filaments while new thin filament / Z-line complexes form at the site that was previously occupied by the M-line. Importantly, if the incorporated proteins are NSPs, then it will result in the formation of an NSP hot spot that is confined to the adjacent inner halves in a pair of in-series sarcomeres (Fig. 8C). An example of this is shown in Supplemental Figure S10C, and hot spots that displayed this type of morphology were classified as H-zone splits.

During the development of our classification procedure, it became evident that a subset of the qualifying NSP hot spots did not align with any of the previously described models of in-series sarcomerogenesis. As such, we created two additional classes: “Short – Atypical” and “Long – Atypical”. Short – Atypical hot spots were defined as being less than two in-series sarcomeres in length, and they typically spanned either ~1/2 sarcomere (Fig. S10D) or ~1.5 sarcomeres (Fig. S10E). In contrast, Long – Atypical hot spots were defined as exceeding two in-series sarcomeres in length, and they were typically composed of ≥3 in-series sarcomeres (Fig. S10F).

After 15 - 30 qualifying NSP hot spots per fiber had been classified, the percentage within each class was calculated, and the per-fiber results were reported in the final datasets.

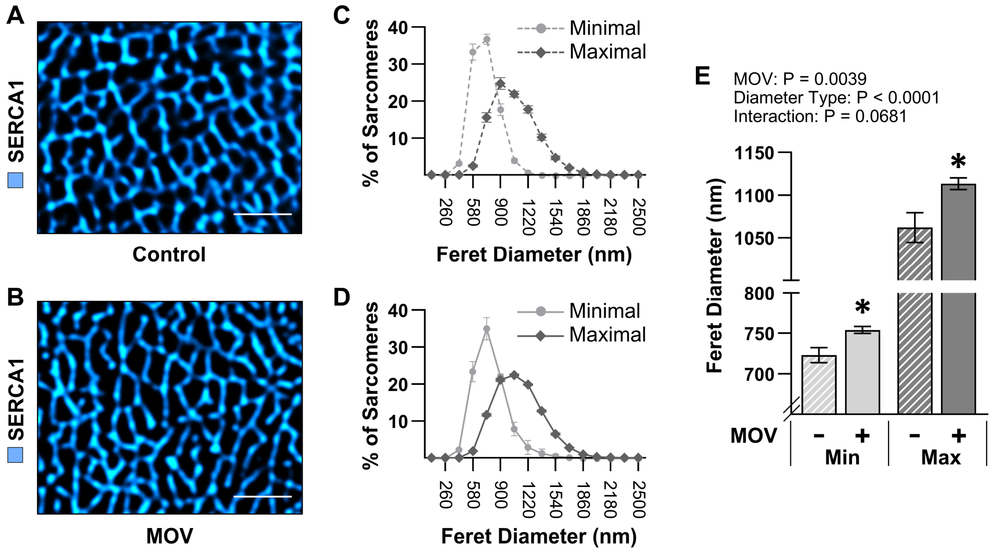

Fig. S1. The Effect of MOV on Sarcomere Diameter. C57BL/6J mice were subjected to a mechanical overload (MOV) or sham (control) surgery. (**A**-**B**) After 8 days, the plantaris muscles were collected, and then mid-belly cross-sections were subjected to immunohistochemistry for dystrophin to identify the periphery of the muscle fibers (not shown) and SERCA1 to identify the periphery of the sarcomeres (cyan). The minimal and maximal Feret diameters of the sarcomeres within randomly selected fibers were determined as detailed in the methods section. Scale bar = 2 µm. (**C**-**D**) Frequency distribution of the minimal (light gray) and maximal (dark gray) Feret diameters of the sarcomeres in the control (**C**) and MOV (**D**) muscles. For the control condition, n = 37868 sarcomeres from 4 independent muscles (5330-16329 sarcomeres per muscle). For the MOV condition, n = 100360 sarcomeres from 9 independent muscles (5687-14103 sarcomeres per muscle). (**E**) The mean values for each of the muscles in C -D were analyzed with two-way RM ANOVA. * Significantly different from control within a given Feret type, $P < 0.05$.

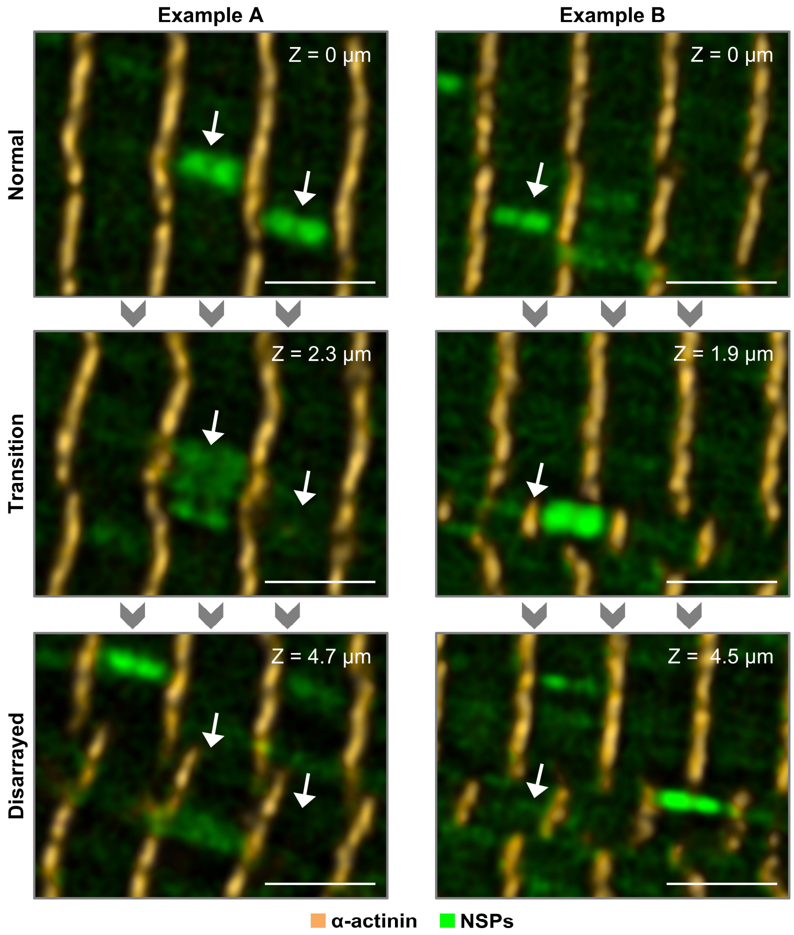

Fig. S2. Three-dimensional Assessment of NSP Hot Spots. MetRS$^{L274G+/+}$ mice were subjected to a mechanical overload (MOV) surgery, and after 7 days, the mice were injected with ANL. The plantaris muscles were collected 24 hr later, and then thick (10 µm) longitudinal sections were subjected to immunohistochemistry for α-actinin (gold) and a click reaction with alkyne-AZDye 555 (green) to label the newly synthesized proteins (NSPs). A representative region of interest was identified and then imaged at the indicated depths of the Z-plane. Arrows point to the same location along the X and Y planes. Two examples are provided to illustrate how NSP hot spots in a region with a "normal" Z-line configuration reside immediately superficial to a region of "disarray". Scale bars = 3 µm.

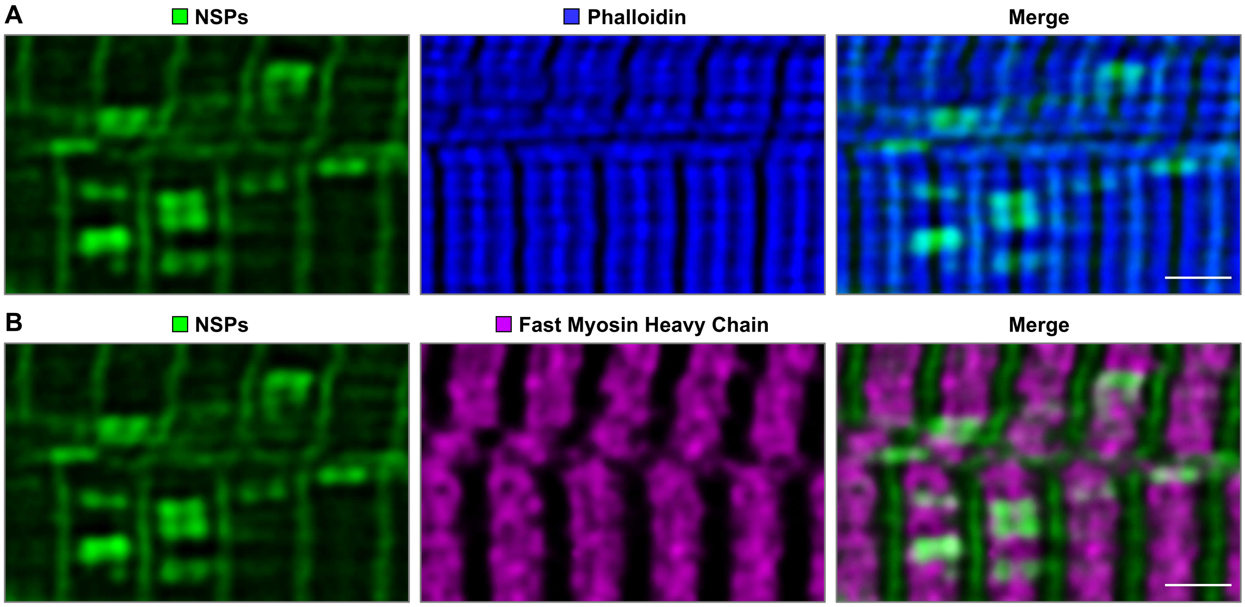

Fig. S3. NSP Hot Spots Align with the Presence of Sarcomeric Proteins. MetRS$^{L274G+/+}$ mice were subjected to a mechanical overload surgery, and after 7 days, the mice were injected with ANL and collected 24 hr later. Representative images from longitudinal sections that had been subjected to click reaction with alkyne-AZDye 555 (green) to label the newly synthesized proteins (NSPs), stained with phalloidin (blue) to label filamentous actin, and subjected to immunohistochemistry to label the fast isoforms of myosin heavy chain (magenta). (**A**) The signals for the NSPs and phalloidin are shown individually and then merged to illustrate that NSP hot spots consistently align with the presence of striated filamentous actin. (**B**) The signals for the NSPs and fast myosin heavy chain are shown individually and then merged to illustrate that NSP hot spots consistently align with the presence of the fast myosin heavy chains. Scale bars = 2 µm.

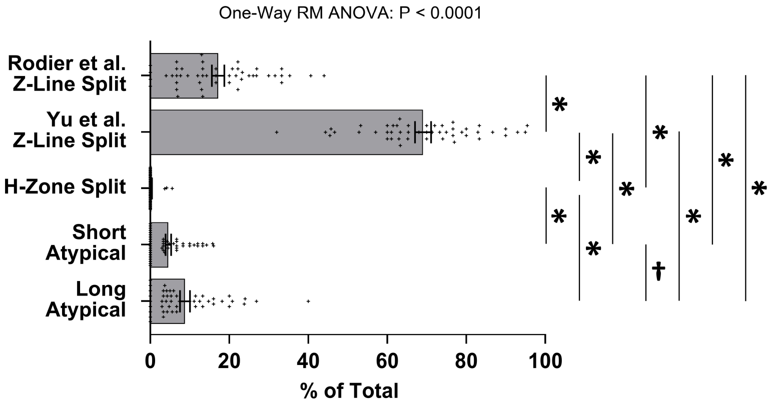

Fig. S4. Distribution of sarcomere-level NSP hot spot morphologies in muscles subjected to mechanical overload. MetRS$^{L274G+/+}$ mice were subjected to a mechanical overload (MOV), and after 7 days, the mice were injected with ANL. The plantaris muscles were collected 24 hr later, and longitudinal sections were subjected to immunohistochemistry for α-actinin and a click reaction with alkyne-AZDye 555 to label the NSPs. The images were assessed for the presence of fibers that had qualifying NSP hot spots (e.g., signal intensity at least 2-fold higher than the local background and dimensions that were consistent those of a sarcomere) and, for each analyzed fiber, the proportion of the NSP hot spots whose morphology aligned with the models of in-series sarcomerogenesis or one of the two atypical split types was determined, n = 48 fibers from 15-30 NSP hot spots / fiber, 10-15 fibers / muscle, 3 muscles (see Supplemental Text for details). Data are presented as individual fiber values as well as group means ± SEM. The data were analyzed with one-way RM ANOVA. * Significant difference between the indicated groups, † $P < 0.05$, * $P < 0.001$.

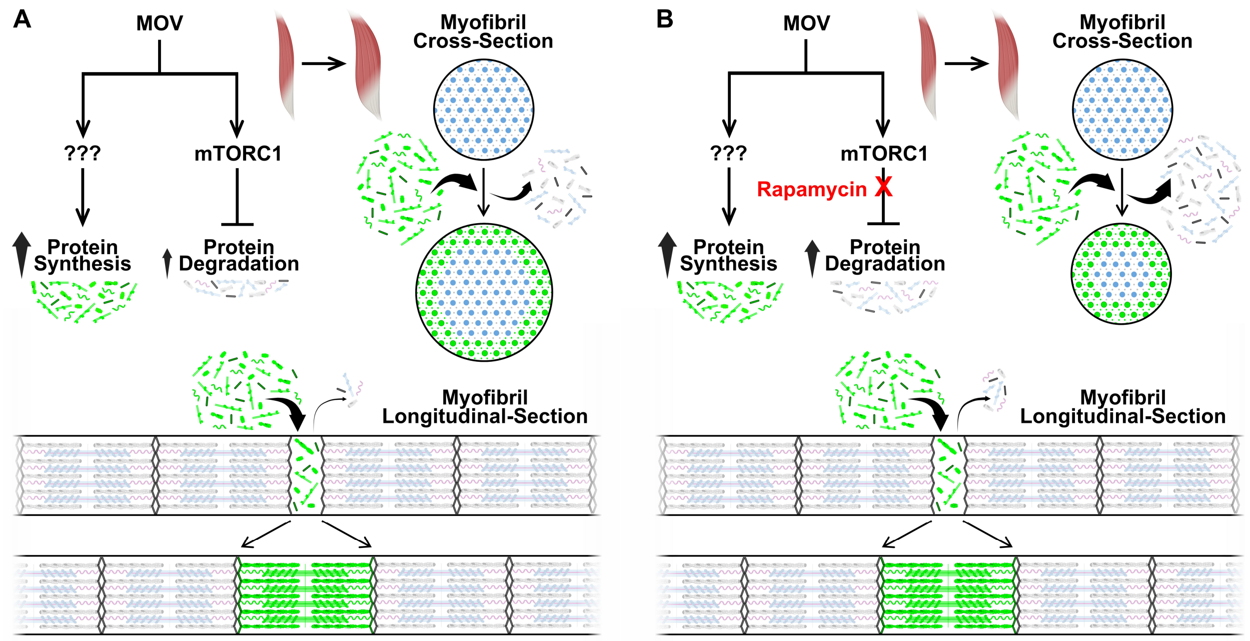

Fig. S5. Illustration of how the Inhibition of mTORC1 Could Differentially Regulate Radial and Longitudinal Growth. The hypothetical model shows plantaris muscles along with cross-sectional and longitudinal views of myofibrils that are composed of pre-existing proteins such as Z-lines (dark gray), titin (light pink), thick filaments (light blue), and thin filaments (gray), as well as newly synthesized proteins (green). (**A**) The hypothetical model predicts that under normal conditions, mechanical overload (MOV) induces a robust increase in protein synthesis through a currently unknown mechanism. MOV also induces robust activation of mTORC1, and this exerts a suppressive effect on protein degradation. As a result, the rate of protein synthesis greatly exceeds the rate of protein degradation, and this leads to a large increase in muscle mass. At the ultrastructural level, the increase in muscle mass can be attributed to an increase in both the diameter (i.e., radial growth) and length (i.e., longitudinal growth) of the myofibrils. Importantly, in the model, it is assumed that the radial growth results from a moderate net increase in the synthesis of new proteins relative to the degradation of the pre-existing proteins. On the other hand, the longitudinal growth is mediated by the in-series addition of sarcomeres, which is almost entirely driven by the synthesis of new proteins (i.e., very few pre-existing proteins are degraded during this process). (**B**) When signaling through mTORC1 is inhibited (e.g., by rapamycin), the MOV-induced increase in protein synthesis is unaffected, but the suppressive effect of mTORC1 on protein degradation is lost. As a result, the overall rate of protein synthesis only moderately exceeds the rate of protein degradation, and thus, the resulting increase in muscle mass is reduced when compared with what occurs in the normal condition. At the ultrastructural level, the smaller increase in muscle mass can be attributed to the loss of radial growth. Specifically, the enhanced degradation of the pre-existing proteins offsets the accumulation of the newly synthesized proteins and thus, the diameter of the myofibrils does not change. Conversely, the degradation of pre-existing proteins does not make a major contribution to the process via which new in-series sarcomeres are added. As such, the increase in protein degradation would exert little, if any, effect on the induction of longitudinal growth.

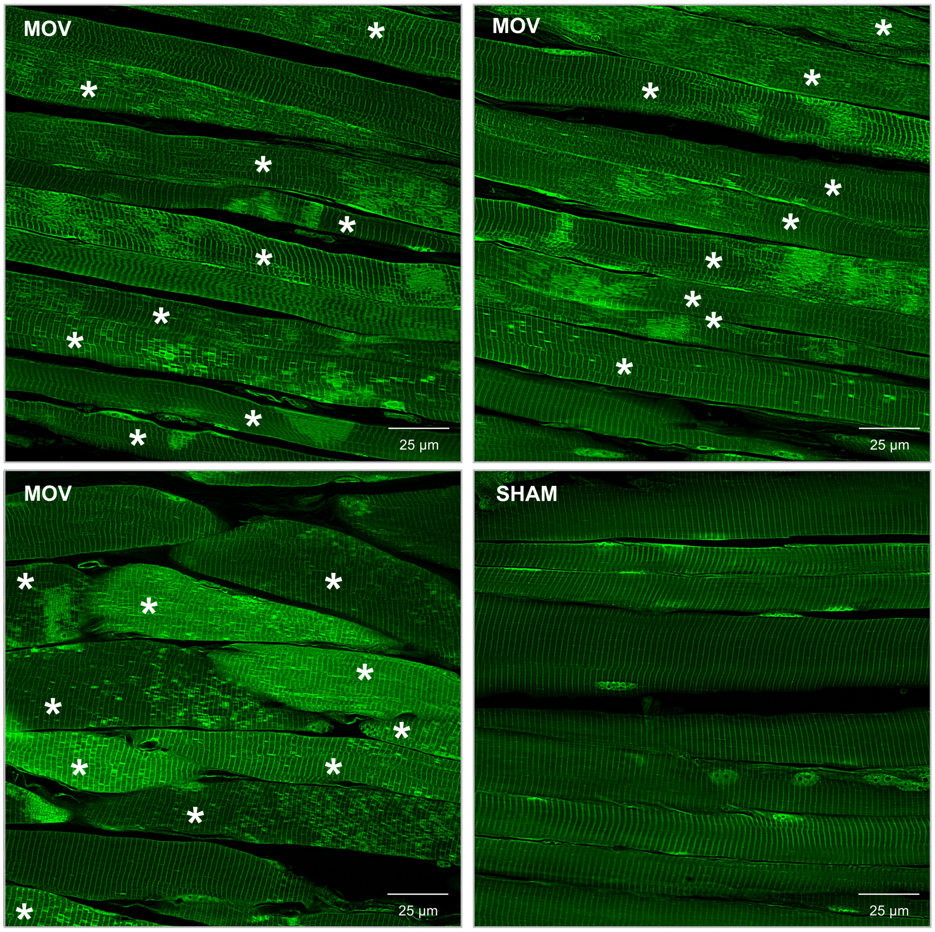

Fig. S6. Identification of NSP Hot Spot Positive Muscle Fibers. MetRS$^{L274G+/+}$ mice were subjected to a mechanical overload (MOV) of the sham surgery, and after 7 days, the mice were injected with ANL and collected 24 hr later. Longitudinal sections were subjected to a click reaction with alkyne-AZDye 555 (green) to label the newly synthesized proteins (NSPs). Muscle fibers containing multiple loci that were densely populated with NSPs were categorized as positive for NSP hot spots. Multiple examples are shown in which NSP hot spot positive fibers are indicated with an asterisk.

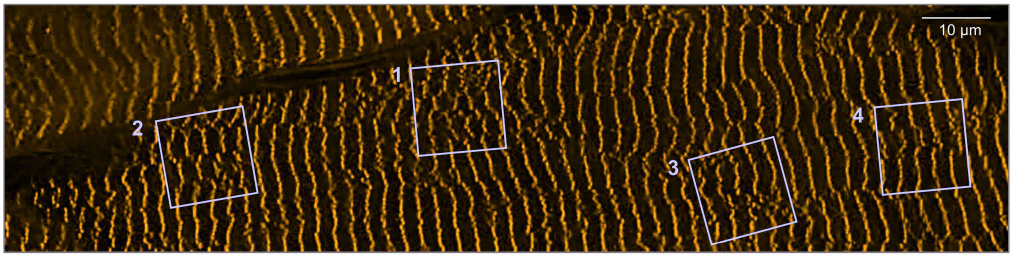

Fig. S7. Selection of "Disarrayed" ROIs in Muscle Fibers Containing NSP Hot Spots. MetRS$^{L274G+/+}$ mice were subjected to mechanical overload, and after 7 days, the mice were injected with ANL. The plantaris muscles were collected 24 hr later, and then longitudinal sections were subjected to immunohistochemistry for α-actinin (gold) and a click reaction with alkyne-AZDye 555 (green) to label the newly synthesized proteins (NSPs) as in Supplemental Figure S6. Muscle fibers that had been classified as positive for NSP hot spots were further evaluated for the presence of ROIs with disarray in the signal for α-actinin. Four representative ROIs are shown and were ranked as having the greatest (**1**) to the least (**4**) amount of disarray.

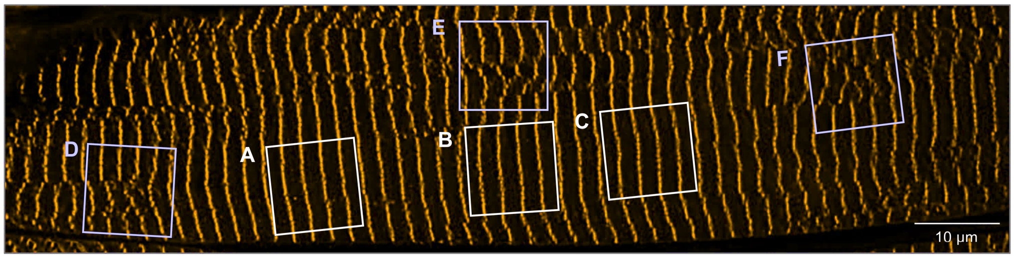

Fig. S8. Selection of "Normal" ROIs in Muscle Fibers Containing NSP Hot Spots. MetRS$^{L274G+/+}$ mice were subjected to mechanical overload, and after 7 days, the mice were injected with ANL. The plantaris muscles were collected 24 hr later, and then longitudinal sections were subjected to immunohistochemistry for α-actinin (gold) and a click reaction with alkyne-AZDye 555 (green) to label the newly synthesized proteins (NSPs) as in Supplemental Figure S6. Muscle fibers that had been classified as positive for NSPs hot spots were further evaluated for the presence of "normal" (**A-C**) and "disarrayed" (**D-F**) ROIs in the signal for α-actinin. When selecting "normal" ROIs for analysis, efforts were made to maximize the distance of the selected ROI from surrounding areas of disarray. In this example, the top-ranking "normal" ROI is A, followed by C, and then B.

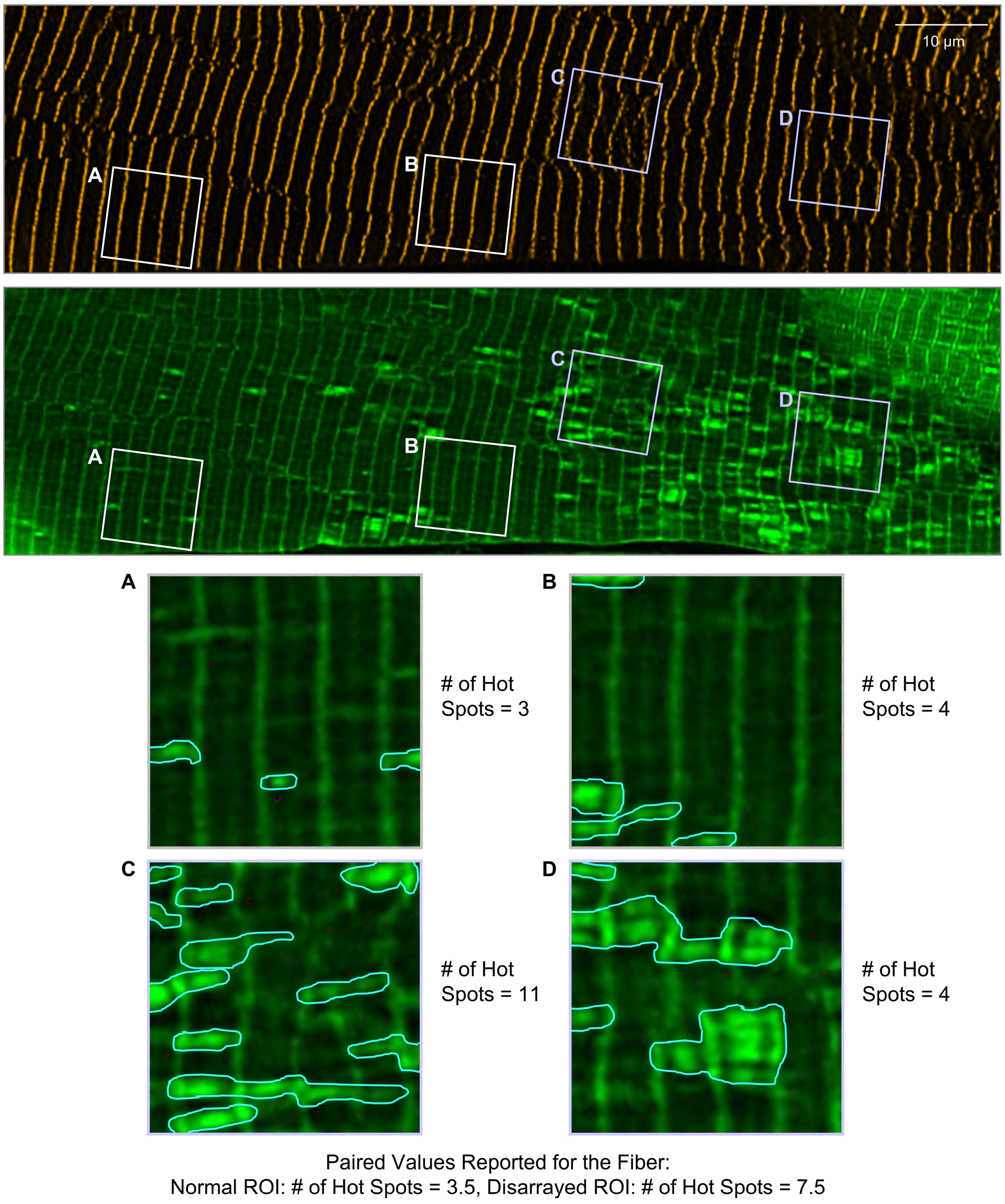

Fig. S9. NSP Hot Spot Quantification in "Normal" and "Disarrayed" Regions. MetRS$^{L274G+/+}$ mice were subjected to mechanical overload, and after 7 days, the mice were injected with ANL. The plantaris muscles were collected 24 hr later, and then longitudinal sections were subjected to immunohistochemistry for α-actinin (gold) and a click reaction with alkyne-AZDye 555 (green) to label the newly synthesized proteins (NSPs). Plantaris muscle fibers that contained both "normal" and "disarrayed" ROIs in the signal for α-actinin were further evaluated to quantify the number of NSP hot spots per ROI. In this example, the number of NSP hot spots in the "normal" (**A-B**) and "disarrayed" (**C-D**) ROIs was averaged and then recorded as paired values for the fiber.

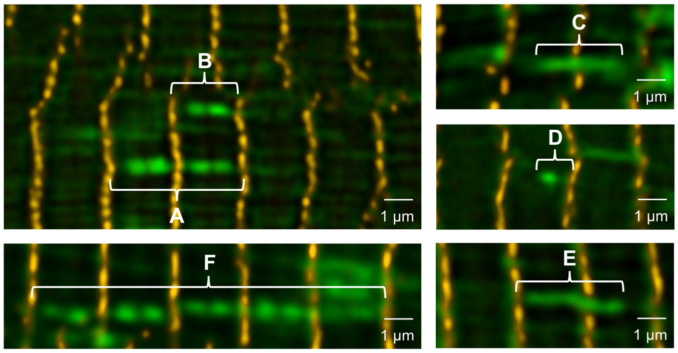

Fig. S10. Examples of the Different Classes of NSP Hot Spots. MetRS$^{L274G+/+}$ mice were subjected to mechanical overload, and after 7 days, the mice were injected with ANL. The plantaris muscles were collected 24 hr later, and then longitudinal sections were subjected to immunohistochemistry for α-actinin (gold) and a click reaction with alkyne-AZDye 555 (green) to label the newly synthesized proteins (NSPs). Examples of the five different classes of NSP hot spots that were quantified in this study are shown. (**A**) Rodier et al. *(42)* Z-line split. (**B**) Yu et al. *(35)* Z-line split. (**C**) H-zone split. (**D-E**) Short – Atypical. (**F**) Long – Atypical.

Target	Sequence (5' → 3')
CMV-CRE Forward Primer (JAX oIMR1084)	GCG GTC TGG CAG TAA AAA CTA TC
CMV-CRE Reverse Primer (JAX oIMR1085)	GTG AAA CAG CAT TGC TGT CAC TT
Wild-type (intact Rosa26) Forward Primer (JAX 26209)	CTG GCT TCT GAG GAC CG
Wild-type (intact Rosa26) Reverse Primer (JAX oIMR9021)	CCG AAA ATC TGT GGG AAG TC
MetRS^{L274G} Mutant Forward Primer (JAX 12614)	ACC ACT ACC AGC AGA ACA CC
MetRS^{L274G} Mutant Reverse Primer (JAX 26209)	GGC AGA TTG CAC TAG CAG AG

Table S1. Genotyping Primers

Movie S1. A pan through the Z-plane of a 10 µm thick longitudinal section of a plantaris muscle that had been subjected to 8 days of MOV and labelled with ANL to visualize the accumulation of NSPs. Due to the section thickness, the NSP hot spots are not sharply resolved. Nonetheless, the video shows many examples of NSP hot spots that appear to occur at an arrayed region but are actually localized immediately superficial or deep to a site of disarray.

Movie S2. A pan through the Z-plane of a 10 µm thick longitudinal section of a plantaris muscle that had been subjected to 8 days of MOV and labelled with ANL to visualize the accumulation of NSPs. Due to the section thickness, the NSP hot spots are not sharply resolved. Nonetheless, the video shows many examples of NSP hot spots that appear to occur at an arrayed region but are actually localized immediately superficial or deep to a site of disarray.

Data S1. The source data for all of the main and supplemental figures is available at Dryad as indicated the in 'Data and Materials Availability' section of the manuscript. The data are provided in an excel file and, where applicable, the formulas for converting raw values into the relative values have been included so that readers can determine how the values in the manuscript were derived.
